# Isolate Removal Methods and Methicillin-resistant *Staphylococcus aureus* Surveillance

**DOI:** 10.3201/eid1110.050162

**Published:** 2005-10

**Authors:** Fenfang Li, Tracy L. Ayers, Sarah Y. Park, F. DeWolfe Miller, Ralph MacFadden, Michele Nakata, Myra Ching Lee, Paul V. Effler

**Affiliations:** * University of Hawaii School of Medicine, Honolulu, Hawaii, USA; † Hawaii Department of Health, Honolulu, Hawaii, USA

**Keywords:** NCCLS, methicillin-resistant Staphylococcus aureus, surveillance, antimicrobial drug resistance, research

## Abstract

The effect of duplicate isolate removal strategies on *Staphylococcal aureus* susceptibility to oxacillin was compared by using antimicrobial test results for 14,595 isolates from statewide surveillance in Hawaii in 2002. No removal was compared to most resistant and most susceptible methods at 365 days and to the National Committee for Clinical Laboratory Standards (NCCLS) and Cerner algorithms at 3-, 10-, 30-, 90-, and 365-day analysis periods. Overall, no removal produced the lowest estimates of susceptibility. Estimates with either NCCLS or Cerner differed by <2% when the analysis period was the same; with either method, the difference observed between a 90- and a 365-day period was <1%. The effect of duplicate isolate removal was greater for inpatient than outpatient settings. Considering the ease of implementation and comparability of results, we recommend using the first isolate of a given species per patient to calculate susceptibility frequencies for *S. aureus* to oxacillin.

## Abstract 2

The first *Staphylococcus aureus* isolate from a patient should be used to calculate oxacillin susceptibility frequency.

Surveillance of antimicrobial susceptibility is critical for developing strategies to control increasing antimicrobial resistance. Aggregation of institutional antibiograms is commonly proposed as a useful means of monitoring antimicrobial resistance trends in a population (*1**–**3*). However, inconsistencies in the methods used to generate antibiogram susceptibility reports, particularly with regard to duplicate isolate removal, make comparing data from different facilities problematic (*2**,**4**–**6*).

To address this situation, in 2002, the National Committee for Clinical Laboratory Standards (NCCLS, currently known as the Clinical and Laboratory Standards Institute) recommended using antimicrobial test results from the first species isolate per patient, per period of data analysis, to calculate susceptibility frequencies (*7*). Other approaches currently in use include not removing any isolates, counting only the most susceptible or most resistant isolate from a patient per surveillance period, and applying the Cerner laboratory management system, a widely used software program (*4*).

Studies comparing the potential effect of using different methods for duplicate isolate removal are limited, i.e., most existing analyses are based on data from a single facility or compared only a few of the many different options for duplicate isolate removal (*4**–**6*). We evaluated the effects of 13 distinct duplicate isolate removal strategies on *Staphylococcus aureus* susceptibility to oxacillin by using antimicrobial susceptibility test results from a statewide antimicrobial resistance surveillance system in Hawaii.

## Methods and Materials

### Data Collection

All available susceptibility data for *S. aureus* isolates identified in Hawaii in 2002 were collected retrospectively from the laboratory information systems of participating facilities and transferred to the State of Hawaii Antimicrobial Resistance Project (SHARP) database. The SHARP system consists of laboratory data from 2 large commercial clinical laboratories and most acute-care hospitals. The 2 commercial laboratories serve most of Hawaii's population (N = 1,211,537) by providing susceptibility testing services for >85% of all nonhospital outpatient settings in Hawaii and performing susceptibility testing for 18 of the 24 acute-care hospitals in the state (*8**,**9*). The remaining 6 acute-care hospitals each maintain their own laboratory to perform susceptibility testing for their respective facility. Susceptibility results from 3 of these hospitals were incorporated into the SHARP database, yielding a final dataset that encompasses 21 (88%) of Hawaii's 24 acute-care hospitals. A review of antibiograms produced by all laboratories in Hawaii in 2001 indicates that the data sources used in the current analysis capture >90% of all *S. aureus* identified in our state annually.

Laboratories participating in SHARP provide isolate-level data, including the specimen collection date, source (e.g., blood, urine, and cerebrospinal fluid), susceptibility test methods (e.g., Kirby-Bauer), and susceptibility test results. Limited demographic patient information is also included in the record, e.g., date of birth and sex, but detailed clinical histories and patient names are not available. The susceptibility testing method used by all laboratories during the study period was the Vitek automatic system, supported by the Kirby-Bauer disk diffusion method (*10*). NCCLS criteria were used to interpret inhibitory zone diameters and MIC. Determination of methicillin susceptibility is based on oxacillin susceptibility testing. The breakpoint for oxacillin resistance was MIC >4 μg/mL or a zone diameter <10 mm. The breakpoint for intermediate isolates was MIC 2–4 μg/mL or zone diameter 11–12 mm. The breakpoint for susceptible isolates was MIC <2 μg/mL or zone diameter >13mm.

For this analysis, *S. aureus* isolates from inpatients in intensive care units (ICUs), other inpatient settings (non-ICU), and outpatient settings (e.g., physician offices, community health centers, hospital outpatients, and emergency departments) were included. Isolates from patients in long-term care homes and prisons were excluded.

### Detection of Duplicate Isolates

Duplicate isolates were identified by using Microsoft Access (Microsoft Corp., Redmond, WA, USA) to sort susceptibility data based on the patient's unique medical record number (MRN), if available, or an assigned patient identifier (APID). When an MRN was not available, the APID was created from the patient's date of birth, sex, reporting laboratory, and identity of the hospital facility or private physician who ordered the culture. The ability of the APID to uniquely identify patients was assessed by generating an APID for the subset of patients who also had MRNs. The corresponding APID was found to be unique for 99% of the records with a unique MRN and assessed to be an acceptable surrogate. The potential effect of using the APID in lieu of the MRN was assessed in a subanalysis that compared the results for records containing an MRN to those from records identified with the APID.

### Methods for Duplicate Isolate Removal

Antimicrobial susceptibility frequencies were calculated by using each of the 5 duplicate isolate removal methods described. For the "no removal" method, susceptibility results for all *S. aureus* isolates in the 2002 database were included in the estimation of the proportion of isolates. For the "most resistant" method, during a 365-day period, irrespective of the number of positive cultures, each patient was counted only once. For any given patient, if a resistant isolate was identified, the first resistant isolate identified was included in the analysis, and all other results, susceptible or resistant, were censored. If no resistant isolates were identified for a patient during the period, the first sensitive isolate was included in the analysis. For the "most susceptible" method, during a 365-day period, irrespective of the number of positive cultures, each patient was counted only once. For any given patient, if a susceptible isolate was identified, the first susceptible isolate identified was included in the analysis, and all other results, susceptible or resistant, were excluded. If no susceptible isolates were identified for a patient during the period, the first resistant isolate was included in the analysis. For the NCCLS method, the susceptibility results for the first *S. aureus* isolate per patient per analysis period, irrespective of body site, antimicrobial susceptibility profile, or other phenotypic characteristics (e.g., biotype), were included in the analysis (*7*). We applied NCCLS criteria for 5 different surveillance periods: 3, 10, 30, 90, and 365 days. Finally, for the Cerner method, a duplicate isolate was defined as from the same patient, same species, and same NCCLS susceptibility category to an individual antimicrobial agent as an immediately previous isolate (*4*). For this study, the Cerner method was modified to include surveillance periods commonly used with other duplicate isolate removal methods: 3, 10, 30, 90, and 365 days. Therefore, in our setting, duplicate *S. aureus* isolates were defined by the modified Cerner methods as the same patient and same susceptibility to oxacillin as the immediately previous isolate found during the same analysis period. Any isolate obtained from a given patient during the period of analysis that showed a change in susceptibility from that of the previous isolate was included in calculations of susceptibility.

For each method, the percentage of susceptible isolates was calculated by dividing the number of susceptible isolates by the number of total isolates eligible for the inclusion method in each particular analysis. Tables 1 and 2 show how the various strategies are applied to hypothetical patient isolates and illustrate how the susceptibility percentages were calculated for each scenario. Ninety-five percent confidence intervals (CIs) for proportions of susceptibility were calculated by using the binomial method. When susceptibility proportions for specific clinical settings (or institutions) were calculated, only isolates obtained in that particular setting (or institution) were eligible for analysis.

**Table 1:** Hypothetical data for Staphylococcus aureus susceptibility to oxacillin*

Patient	Day						
	1	2	4	11	20	31	100
1	R	S	R	R	S	S	S
2	R	R	R	R	R	R	R
3	S	S	S	S	S	R	R

*R, resistant; S, susceptible.

**Table 2:** Application of different methods of duplicate isolate removal based on hypothetical data in Table 1*

Method	No. isolates	No. susceptible (%)†
No removal	21	9 (43)
Cerner, 3 d	19	8 (42)
NCCLS, 3 d	18	7 (39)
Cerner, 10 d	15	6 (40)
NCCLS, 10 d	12	4 (33)
Cerner, 30 d	12	5 (42)
NCCLS, 30 d	9	3 (33)
Cerner, 90 d	10	4 (40)
NCCLS, 90 d	6	2 (33)
Cerner, 365 d	7	3 (43)
NCCLS, 365 d	3	1 (33)
Most resistant	3	0
Most susceptible	3	2 (67)

*d, days; NCCLS, National Committee for Clinical Laboratory Standards. †Susceptibility percentage is calculated as the proportion of the number of susceptible isolates divided by the number of total isolates tested and eligible for inclusion according to the analysis method used.

## Results

Susceptibility testing results were identified for 14,595 *S. aureus* clinical isolates obtained from 10,892 patients. A total of 3,725 isolates were from 2,749 patients with an associated MRN; 10,870 were from 8,143 patients identified with an APID. For all patients, the isolate-to-patient ratio was 1.3.

**Figure 1:**
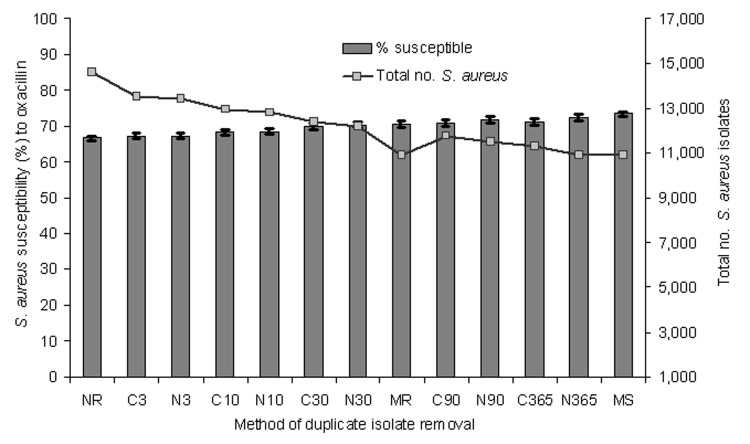
Effect of duplicate isolate removal strategies on the number of Staphylococcus aureus isolates and percentage susceptible to oxacillin for all patients in Hawaii, 2002. The 95% confidence interval for the proportion is shown in brackets. NR, no removal; MR, most resistant; MS, most susceptible; N, NCCLS algorithm; C, Cerner algorithm; the number indicates the days in the analysis period.

Figure 1 depicts the effect of duplicate isolate removal on *S. aureus* susceptibility to oxacillin for all isolates. NCCLS and Cerner methods produced similar estimates of susceptibility for any given analysis period, i.e., the difference between the 2 methods was insignificant. Furthermore, the difference in susceptibility percentage between a 90-day and 365-day period was <1% by either NCCLS or Cerner criteria, which was insignificant. With both Cerner and NCCLS methods, the general trend for estimates of susceptibility increased as the period of analysis lengthened.

No removal resulted in the lowest susceptibility estimate (67%) observed, even lower than that for most resistant (Figure 1). Overall, an inverse relationship was observed between number of isolates included in the analysis and proportion of susceptible isolates (Figure 1). For both Cerner and NCCLS methods, point estimates of susceptibility rose slightly as the period of analysis increased and the number of isolates included in the susceptibility calculations decreased.

The patterns observed for all isolates combined remained unchanged when stratified by different clinical settings, i.e., ICU, non-ICU, and outpatient (Table 3). Within a given clinical setting, the difference in susceptibility frequencies with the 90- and 365-day intervals by either Cerner or NCCLS was <1%.

**Table 3:** Effect of duplicate isolate removal on Staphylococcus aureus susceptibility to oxacillin, by clinical setting*

Method	ICU		Non-ICU		Outpatient	
	No. isolates	No. susceptible (%, 95% CI)	No. isolates	No. susceptible (%, 95% CI)	No. isolates	No. susceptible (%, 95% CI)
No removal	843	465 (55, 52–59)	3,894	1,971 (51, 49–52)	9,858	7,281 (74, 73–75)
Cerner, 3 d	712	387 (54, 51–58)	3,363	1,705 (51, 49–52)	9,590	7,101 (74, 73–75)
NCCLS, 3 d	708	384 (54, 51–58)	3,328	1,682 (51, 49–52)	9,559	7,078 (74, 73–75)
Cerner, 10 d	629	355 (56, 53–60)	3,090	1,614 (52, 50–54)	9,500	7,045 (74, 73–75)
NCCLS, 10 d	616	352 (57, 53–61)	3,038	1,584 (52, 50–54)	9,461	7,020 (74, 73–75)
Cerner, 30 d	589	345 (59, 55–63)	2,849	1,569 (55, 53–57)	9,280	6,907 (74, 74–75)
NCCLS, 30 d	574	341 (59, 55–63)	2,772	1,534 (55, 53–57)	9,222	6,875 (75, 74–75)
Most resistant	545	317 (58, 54–62)	2,426	1,355 (56, 54–58)	8,427	6,295 (75, 74–76)
Cerner, 90 d	574	339 (59, 55–63)	2,681	1,525 (57, 55–59)	8,905	6,667 (75, 74–76)
NCCLS, 90 d	558	335 (60, 56–64)	2,563	1,480 (58, 56–60)	8,802	6,617 (75, 74–76)
Cerner, 365 d	564	336 (60, 56–64)	2,578	1,485 (58, 56–60)	8,589	6,444 (75, 74–76)
NCCLS, 365 d	545	332 (61, 57–65)	2,426	1,420 (59, 57–60)	8,427	6,368 (76, 75–76)
Most susceptible	545	334 (61, 57–65)	2,426	1,468 (61, 59–62)	8,427	6,433 (76, 75–77)

*d, days; ICU, intensive care unit; NCCLS, National Committee for Clinical Laboratory Standards; CI, confidence interval.

Differences in the magnitude of the effect of duplicate isolate removal were observed across different clinical settings (Table 3). For example, the effect of removal in the non-ICU and ICU environments was an increase of 8% and 6%, respectively, in the susceptible proportion when comparing 365-day NCCLS results to no removal; this comparison resulted in an increase of 2% in the outpatient setting.

In a subanalysis restricted to the 2,749 patients (3,725 isolates) with an associated MRN, results were highly analogous to those observed for the larger cohort as a whole. Specifically, for each clinical setting, the difference in the susceptibility estimate between NCCLS and Cerner methods was insignificant for any given period of analysis, and the difference in susceptibility percentage between a 90- and 365-day time period was <1% with either NCCLS or Cerner. In addition, the outpatient setting continued to show the least effect of duplicate isolate removal when compared to inpatient settings.

**Figure 2:**
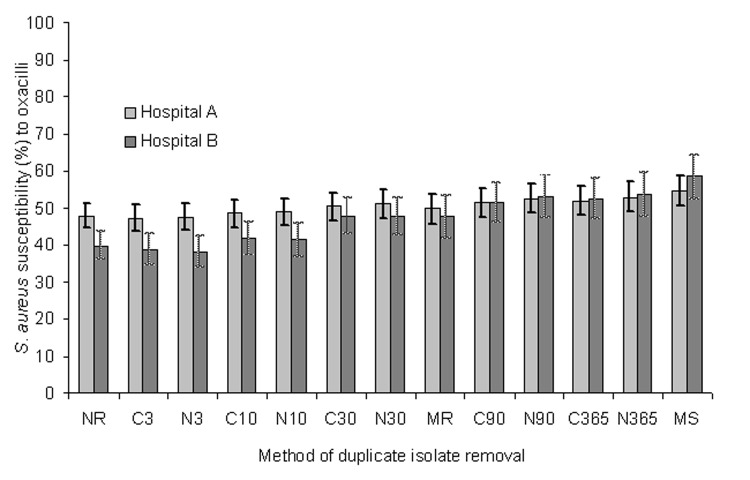
Effect of duplicate isolate removal strategies on the number of Staphylococcus aureus isolates and percentage susceptible to oxacillin for all patients in Hawaii, 2002. The 95% confidence interval for the proportion is shown in brackets. NR, no removal; MR, most resistant; MS, most susceptible; N, NCCLS algorithm; C, Cerner algorithm; the number indicates the days in the analysis period.

Finally, we examined the effect of each deduplication strategy for inpatients (ICU and non-ICU) at major hospitals. Although the hospitals were of different sizes, and their respective rates of MRSA differed by what was seen with no removal, the effects of deduplication observed for each hospital individually were similar to those observed for the population-based surveillance dataset as a whole. Figure 2 illustrates the results of isolate deduplication from 2 of the hospitals with the largest number of *S. aureus* isolates in 2002.

## Discussion

To our knowledge, this report is the first to compare the effect of different deduplication strategies on susceptibility patterns derived from a statewide, population-based antimicrobial resistance surveillance system. The relatively large sample of *S. aureus* isolates in this study was obtained from multiple healthcare settings by a variety of clinical laboratories. Because of the diversity of practice patterns represented in the study, we believe our findings are likely to be applicable to other facilities and agencies conducting antimicrobial resistance surveillance for MRSA.

The major findings from this analysis are the following: 1) NCCLS and modified Cerner methods yield similar results for a given analysis period; 2) with both NCCLS and modified Cerner, the number of total isolates included and the percentage that are MRSA decrease slightly as the period of analysis increases; 3) point estimates of the proportion of MRSA produced for a 90- or 365-day analysis period were statistically similar by either NCCLS or Cerner; and 4) the effect of deduplication was greater for inpatient settings compared to outpatient settings.

We also found that no removal produced the highest estimates of MRSA resistance, even higher than most resistant for the same analysis period. While this finding may seem at first paradoxical, it demonstrates the influence that practice patterns may have on reported rates of MRSA and why deduplication is critical. In our setting, cultures of samples from patients with MRSA were obtained more frequently than cultures from those with methicillin-susceptible strains (MSSA) so that, on average, individual patients with MRSA contributed more isolates, which could be included in the estimation of MRSA rates. This fact also explains why we observed, for both NCCLS and Cerner, an inverse relationship between the length of the analysis period and the rate of MRSA, i.e., the greater number of isolates included with shorter periods of analysis meant more MRSA isolates relative to MSSA strains. If reculturing patient samples is influenced by prior susceptibility testing results, an institution's MRSA percentages might be determined by the practice patterns of physicians working within the institution. Furthermore, trends in antimicrobial resistance could be obscured if practice patterns changed and reculturing samples from MSRA patients became more or less common.

Duplicate isolate removal facilitates comparing data among institutions and monitoring trends over time. However, at present, no clear consensus has been reached on the definition of duplicate isolates, and duplicate isolates cannot be easily identified with certainty in clinical practice (*2**,**4**–**6*). Our study found that NCCLS and modified Cerner methods yield similar results, and for either a 90-day or a 365-day analysis period, the produced estimates fall between results produced by the most resistant and most susceptible methods. Thus, NCCLS and Cerner might both be considered reasonable approaches. However, the NCCLS method has 1 major advantage: NCCLS is the only method that does not require the infection control practitioner to simultaneously compare susceptibility results for multiple isolates obtained from a given patient during the analysis period. With NCCLS, one simply includes the susceptibility results for the first isolate obtained during the analysis period. This straightforward approach would minimize opportunity for error and result in more consistent implementation of the deduplication process (*5*).

Regardless of which deduplication strategy is selected, the question remains which analysis period to adopt (*5**,**6**,**11*). A longer surveillance period increases the probability that an isolate representing a truly new resistance event (rather than a duplicate) will be removed (*11**,**12*). The purpose of antimicrobial resistance surveillance is to assess temporal trends, evaluate intervention efforts, and ultimately improve clinical outcomes on a population-based level. While the treating clinician will need to consider the susceptibility results for each isolate obtained from the patient, and perhaps promptly change therapy in response to new developments (*13**,**14*), population-based recommendations for antimicrobial treatment are not likely to be altered on the basis of 3, 10, or even 30 days of surveillance data. Therefore, adopting either a 90- or 365-day analysis period for MRSA surveillance appears reasonable. At the statewide level in Hawaii, the results seen with the 90- and 365-day NCCLS methods were nearly identical, so either option would be appropriate.

Antibiograms should be individualized for specific clinical areas within an institution (e.g., ICUs) (*15**,**16*). In Hawaii, we observed differences in both MRSA rates as well as the magnitude of the effect of deduplication among the ICU, non-ICU, and outpatient settings; outpatient settings had the least effect. The greater effect of deduplication among inpatients may result from both the higher rate of MRSA among hospitalized patients and a greater likelihood of inpatients, especially those with MRSA, to have samples recultured compared to outpatients.

A major limitation of this analysis is that, because of medical confidentiality issues, we did not have patients' names. Since unique identities were determined by using an MRN generated by the treating facility or medical plan, a patient whose sample was cultured in >1 clinical facility during the analysis period might be miscounted as 2 persons. A related concern is that some laboratories did not provide the patient's MRN; for these patients, we had to use other information to generate an APID. While the APID process was not perfect and a small proportion of persons may have been misclassified as nonunique, a subanalysis that used only records with MRNs produced the same pattern of results as the analysis that used the larger dataset that incorporated the APID. This finding suggests that any misclassifications that resulted from using the APID did not substantially alter the relative effect of the different deduplication strategies we studied.

A second limitation is that we only assessed oxacillin resistance among *S. aureus*, so that conclusions regarding the effect of deduplication on other microorganisms must be made with caution. In addition, our analysis was not stratified by specific anatomic culture site (e.g., blood vs. skin); therefore, the effect of various deduplication strategies on isolates from specific culture sites could not be addressed.

A third limitation is that we did not include deduplication methods that take into account patterns of phenotypic resistance to multiple antimicrobial agents simultaneously (i.e., antibiotypes), as is practiced in some European countries (*12*). Tracking resistance by antibiotypes may show the actual number of infectious events or the selection of resistance occurring within the surveillance period. While these tasks are important for surveillance in some settings, the main purpose of our study was to evaluate a variety of uncomplicated strategies for generating communitywide susceptibility reports to specifically monitor MRSA trends and guide selection of empiric therapy. Nevertheless, further work is needed to examine the role of antibiotype surveillance in population-based antimicrobial surveillance systems.

We conclude that the NCCLS recommendation of including the first isolate of a given species per patient per analysis period, irrespective of body site, antimicrobial susceptibility profile, or other phenotypic characteristics, yielded results similar to other duplicate isolate removal methods and is straightforward in its implementation. Application of the techniques we examined had the same effect regardless of the institution. To aid our understanding of MRSA in both infection control practice and public health, we urge the widespread adoption of an industry standard. We suggest that adopting the 90- or 365-day NCCLS method would be appropriate, taking into account the goals of surveillance and the resources required.
